# Soleus H and Lower Limb Posterior Root Muscle Reflexes During Stepping After Incomplete SCI

**DOI:** 10.3389/fresc.2022.789333

**Published:** 2022-05-13

**Authors:** Keith E. Tansey, Bradley J. Farrell, Joy A. Bruce, William B. McKay

**Affiliations:** ^1^Center for Neuroscience and Neurological Recovery, Methodist Rehabilitation Center, Jackson, MS, United States; ^2^Shepherd Center, Atlanta, GA, United States

**Keywords:** spinal cord injury, robotic stepping, neuromodulation, soleus H reflex, posterior root muscle reflex

## Abstract

The goal of this study was to examine and compare the step cycle related modulation of the soleus H and posterior root muscle (PRM) reflexes in subjects with and without spinal cord injury. Ten subjects without neurological injury and fifteen subjects with spinal cord injury (SCI) underwent soleus H reflex and lower limb PRM reflex testing while standing and stepping in a robotic gait orthosis. Reflex amplitudes were evaluated during standing, mid stance and mid swing to determine if speed and/or injury altered step cycle related neuromodulation. H and PRM reflexes in the soleus underwent step cycle related modulation in injured and uninjured subjects though the degree of modulation differed between the two reflexes with the H reflex showing more step cycle related modulation. We found in the SCI group that both the soleus H and soleus PRM reflex amplitudes were higher relative to the non-injured group and modulated less during the step cycle. We also found that modulation of the soleus H reflex, but not soleus PRM reflex, correlated to the lower extremity motor scores in individuals with SCI. Our evidence suggests that the inability to provide appropriate step cycle related reflex modulation may be due to decreased supra-spinal regulation of motoneuron and spinal excitability and could be an indicator of the severity of injury as it relates to clinically measured lower extremity motor scores.

## Introduction

In humans, standing and walking involves integration of afferent, segmental (interneuronal), and supraspinal inputs onto motoneuron pools leading to patterned muscle activation and deactivation across multiple muscle groups. In animals, this patterned discharge seems to be the primary result of a spinal or central pattern generator (CPG) located within the spinal cord [e.g., (1)] and though humans have some indirect evidence of a CPG (2–4), if it exists, the CPG is weaker and far less responsive to afferent input and more reliant on input from supraspinal centers (5). Thus, after spinal cord injury (SCI), the ability to generate appropriate muscle activation patterns for standing and stepping is often lost.

These muscle activation patterns seen during stepping are often measured using continuous electromyography (EMG) over lower limb muscle groups during over-ground walking or treadmill stepping. However, it is difficult to compare between individuals without neurological injury and those who have suffered a SCI because of large deviations in gait due to differences in speed, reliance on assistive devices, etc. Therefore, standardized walking conditions, such as those imposed by a robotic gait orthosis, may allow better comparisons of activation patterns during stepping at the same speed and body weight support.

In addition to continuous EMG during stepping, soleus H reflexes triggered at different points within the step cycle represent the central effects on sensory input during walking (6, 7) and when stepping in a robotic gait orthosis (8). While tibial nerve stimulation eliciting soleus or gastrocnemii H reflexes allows insight into the triceps surae' s step related modulation, stimulation via transcutaneous spinal stimulation using electrodes over the thoracolumbar enlargement of the spinal cord has demonstrated the ability to generate bilateral reflex responses recordable in major leg muscle groups (9–14). These compound muscle action potentials have similar neurophysiological properties and are referred to as posterior root muscle reflexes [PRM reflexes; (10)], a nomenclature used throughout the rest of the article. These PRM reflexes modulate with the gait cycle (9, 12, 15), are refractory using short interval paired-pulse stimuli (10, 15) and suppressed using vibration and reciprocal inhibitory mechanisms (10). Since these reflexes are expressed across many muscle groups in the lower limb, they may provide additional insight into muscle coordination during voluntary, semi-automatic tasks such as walking in persons with and without incomplete spinal cord injury. However, to our knowledge, the relationship of the soleus H and PRM reflex has not been clearly established and furthermore the modulation of these PRM reflexes has not been evaluated while walking with body weight support after motor incomplete SCI.

Therefore, the objective of this study was to compare modulation of the soleus H reflex and lower limb PRM reflexes during robotic assisted stepping in subjects with and without motor-incomplete spinal cord injury. We hypothesized that the soleus PRM reflex is a close analog to the soleus H reflex and therefore would demonstrate similar modulation across conditions and that after motor incomplete spinal cord injury the ability to appropriately modulate reflex behavior, whether H or PRM reflex, with the step cycle would be diminished.

## Methods

### Study Participants

Fifteen subjects (one female) with motor-incomplete SCI (SCI group) and 10 subjects (three females) without injury [Non-injured (NI) group] participated in the study. Inclusion criteria for the subjects with SCI were as follows: level of injury C4-T10 [International Standards for the Neurological Classification of SCI (ISNCSCI) exam]; motor incomplete [American Spinal Injury Association (ASIA) Impairment Scale (AIS) C or D]; between 18 and 60 years of age; and were at least 3 months post SCI (see Table 1 for demographics). In addition to impairment classified by the AIS grade, lower extremity motor scores (LEMS) were recorded by a trained physical therapist. All subjects with SCI had undergone limited locomotor training upon enrollment (<3 sessions total) or had not received locomotor training for at least 6 months to avoid the confound of any training induced reflex changes. Subjects with SCI had to show evidence of lower extremity voluntary electromyographic (EMG) activity prior to being enrolled in the study (16) and reflex function as assessed by soleus H reflex testing. Participants, with and without SCI, were excluded if they had implanted electronic devices, a coexisting neurological condition (e.g., concomitant traumatic brain injury) or had injuries that precluded them from locomotor training (e.g., orthopedic injuries/limitations). Informed consent was obtained prior to participation in the study and all procedures were approved by the institutional review boards at Shepherd Center and the Atlanta Veterans Affairs Medical Center.

**Table 1:** Demographics and stimulus parameters.

**(A) NI group**								
**ID**		**Sex**		**Age**		**Hmax**		**PRM**
						**intensity**		**matched**
								**intensity**
NI1		F		41		30		190
NI2		M		23		25		156
NI3		F		23		11		105
NI4		M		24		11		218
NI5		M		31		15		180
NI6		M		26		15		115
NI7		M		28		9		215
NI8		F		25		13		205
NI9		M		22		12		155
NI10		M		32		18		388
	NI Mean			28 (6)		16 (7)		193 (79)
**(B) SCI group**								
**ID**	**Sex**	**Age**	**Hmax intensity**	**PRM**	**Time since**	**Strength**	**LOI**	**AIS**
				**matched**	**injury**	**(LEMS)**		
				**intensity**	**(months)**			
SCI01	M	29	41	91.5	20	0	C5	C
SCI02	M	54	44	300	13	3	C4	C
SCI03	M	30	42	360	13	11	C5	C
SCI04	M	46	47	295	6	12	C5	C
SCI05	M	21	58	155	3	21	C8	C
SCI06	M	24	23	240	6	24	C7	C
SCI07	M	28	9.5	135	12	25	C6	C
SCI08	M	52	65	330	3	28	C5-6	C
SCI09	M	49	25.5	262	16	32	C5	D
SCI10	M	47	27	165	25	35	T9	C
SCI11	M	20	34	210	3	35	C4	C
SCI12	M	47	25	145	19	36	T4	D
SCI13	M	22	12	285	34	39	C5	C
SCI14	M	59	28	300	3	41	C3	D
SCI15	F	45	44	235	18	43	T5	D
SCI Mean		38 (14)	35 (16)	234 (83)	13 (9)	24 (13)		

*There was a significant difference between NI and SCI groups Hmax intensity. LEMS, Lower Extremity motor score; AIS, American Spinal Injury Association Impairment Score*.

### Recording and Stimulation Setup

To record the H and PRM reflexes, surface EMG electrodes were placed bilaterally over the hip adductors (Add), rectus femoris (Quadriceps, Quad), biceps femoris (Hamstrings, Ham), tibialis anterior (TA) and soleus (Sol). Prior to placing the recording electrode, the skin was scrubbed with alcohol and a mild abrasive gel and then the electrode was taped down and wrapped with an all-compressive-elastic bandage. The active sensors had medical grade stainless-steel electrode interfaces of two 12-mm disks at an inter-electrode distance of 17 mm and with a pre-amplifier gain of 20 (MA-411, Motion Lab Systems, Inc., Baton Rouge, LA, US). The EMG signals were amplified using the MA-300 system (Motion Lab Systems, Inc., Baton Rouge, LA, US) with a total gain of 350 and the bandwidth from 10 Hz to 1 kHz. If any 60 Hz ambient noise was observed in any EMG channel, the skin there was re-prepped and the EMG electrode re-applied until that signal disappeared.

For H reflex testing, the left side was chosen in all non-injured subjects and in 11/14 SCI subjects. In the other 3 SCI subjects the right side was chosen due to poor reflex stability in the left limb. To elicit a soleus H reflex, stimulating electrodes were placed over the tibial nerve in the popliteal fossa (cathode; 2 cm diameter) and over the proximal portion of the patella (anode; 5 cm diameter). To elicit PRM reflexes, two stimulating electrodes (cathode; 5 cm dia.) were placed on the back to the left and right sides of the T11/T12 spinous processes) and a large reference electrode over the abdomen (anode; 10 × 15 cm). All electrodes were carbon gel electrodes (Empi, St. Paul, MN, USA). Stimuli were delivered using a constant current, monophasic stimulator (DS7A, Digitimer, Hertforshire, UK). Reflex amplitude and EMG activity were continuously monitored via a custom written software program (Labview 14.0, National Instruments). All subjects underwent the same protocol while in the robotic gait orthosis (Lokomat Pro, Hocoma Inc., Zurich, CH) with 60% of their body weight supported (BWS). This level of support was chosen to ensure that all subjects with injury could maintain stepping at the same BWS.

### Standing Reflex Testing

**Figure 1:**
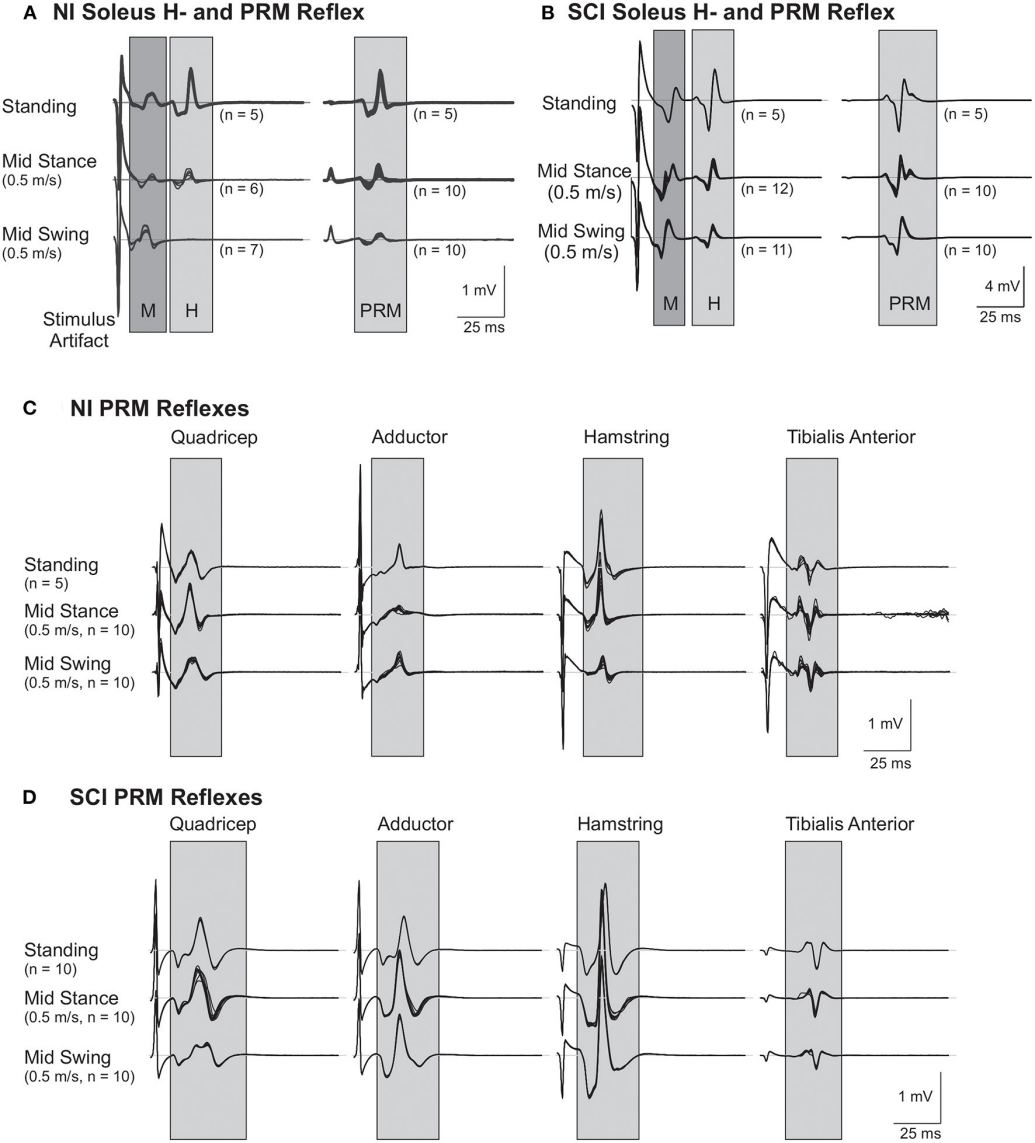
Representative soleus H and lower limb posterior root muscle (PRM) Reflexes. **(A,B)** Soleus H and PRM reflexes from a subject without injury [NI#, **(A)**] and a subject with a spinal cord injury [SCI#, **(B)**]. Note the mid-swing reduction of H and PRM in the soleus is absent in the SCI subject. **(C,D)** PRM reflexes from other lower limb muscles: hip adductor (Adductor), rectus femoris (Quadriceps), biceps femoris anterior (Hamstring), and tibialis anterior from the same subject without injury **(C)** and the same subject with SCI **(D)**. Gray areas highlight the approximate time frame of the H and PRM reflexes.

To examine the soleus H reflex, stimulation intensity was initially set below motor threshold and was slowly increased until the peak-to-peak amplitude of the H reflex reached a maximum during standing in the robotic gait orthosis (Figures 1A,B; representative NI and SCI subject, respectively). A minimum of five H reflexes recorded at this intensity, and at 0.2 Hz to avoid reflex conditioning, determined the mean peak-to-peak M-wave and H reflex amplitude during standing (H_max_). Stimulus intensity was then raised further to determine the maximum soleus M-wave amplitude (M_max_) during standing. Similar procedures were followed to elicit PRM reflexes through trans-abdominal electrodes. Initially, stimulus intensity for PRM reflexes was set below motor threshold and increased slowly until motor threshold was reached for each muscle group and then further increased until the peak-to-peak amplitude of the soleus PRM reflex was within ±10% of standing H_max_ (Figures 1A,B). In one subject (SCI14), PRM reflex amplitude could not be matched to the H_max_ during standing and therefore the highest stimulation intensity that could be comfortably tolerated was utilized for the remainder of the study. At least five PRM reflexes that matched H_max_ during standing were recorded (see Table 1 for H and PRM reflex stimulation intensities). In addition to the soleus standing PRM reflex, we recorded PRM reflexes for the other lower extremity muscles at this corresponding intensity (Figures 1C,D).

### Reflex Testing During Stepping

**Figure 2:**
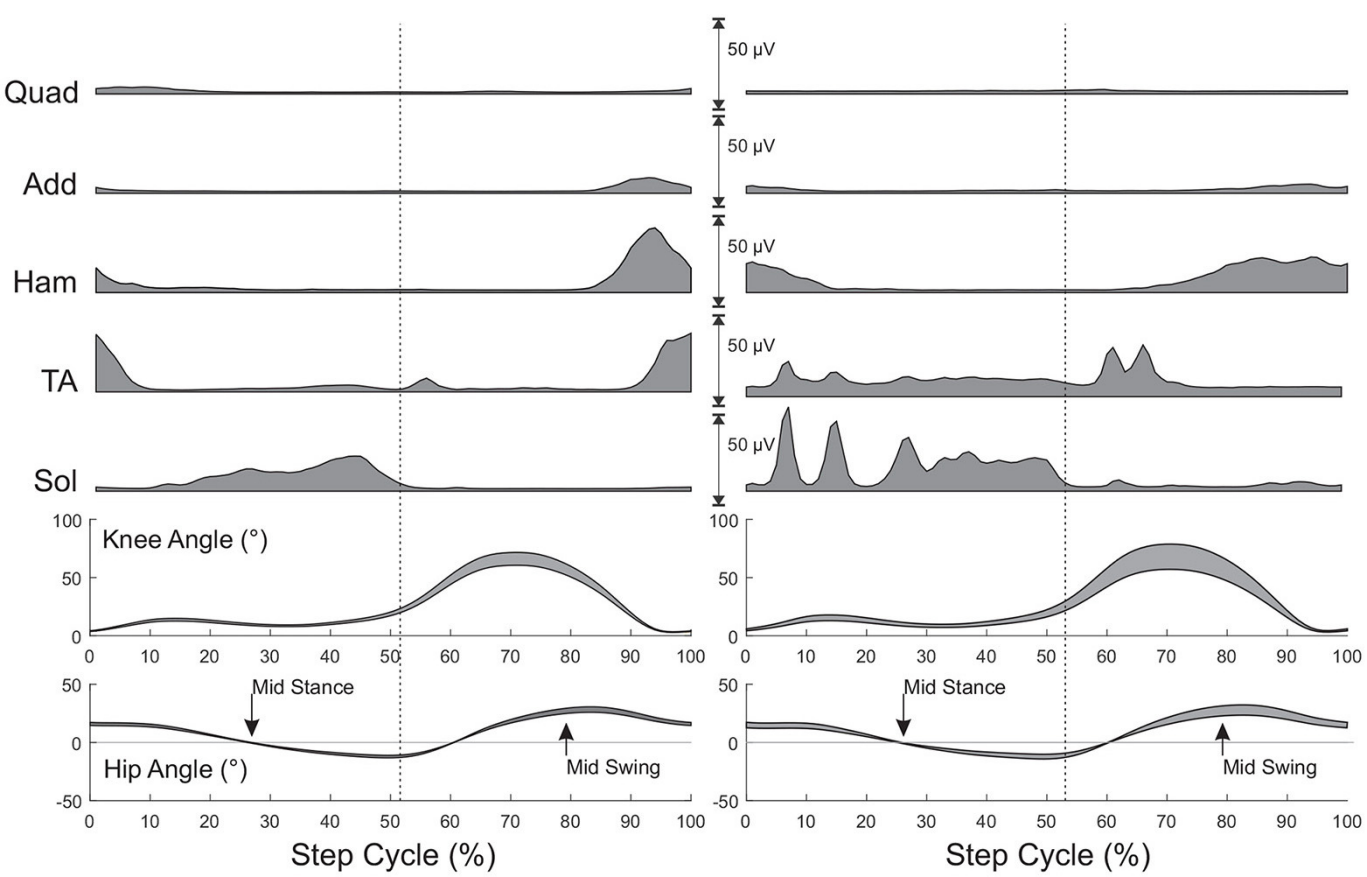
Muscle activity and joint angles during stepping for a subject without injury **(Left)** and a subject with SCI **(Right)**. All values are time normalized to 100% of the step cycle. Muscle activity is the mean activity at each 1% of the step cycle over 25 steps prior to eliciting reflexes. Joint angles represent mean (+SD) during the same steps. Arrows indicate mid stance and mid swing phase of stepping when reflexes would be triggered. Mid stance and mid swing hip joint angles were established during this initial stepping period. Vertical dashed lines represent the transition from stance to swing.

After completing standing reflex testing, all subjects underwent H and PRM reflex testing while stepping at 0.50 m/s and then 0.69 m/s (1.8 and 2.5 kph, respectively) in the robotic gait orthosis. At each speed, soleus H reflex and PRM reflex stimuli were delivered automatically, based on the robotic gait orthosis joint angle output (hip angle equals 0 degrees), first during mid stance phase and then during mid swing phase of the step cycle (Figure 2). For H reflex testing during stepping, stimulus intensity was manually adjusted until the M-wave was within 15% of the measured M-wave elicited during standing (17) and was monitored through real-time updates. For PRM reflex testing during stepping, stimulus intensity was not adjusted from standing as there is no M-wave to match and going from standing to stepping within the support harness should not alter the position of the electrodes relative to the spinal cord. For both H and PRM reflexes, a minimum of 10 stimuli were delivered and recorded every 3rd to 4th step for slow and fast speeds to allow at least 7 s between stimuli to avoid habituation. Subjects were offered water and breaks during the stepping protocol, if needed. The total stepping time for each subject during a recording session varied but was generally <45 min.

### Data Recording and Analysis

Raw EMG signals (including background EMG and reflexes) were recorded at 2 kHz to a computer hard drive continuously throughout the protocol. During post processing, EMG data from each muscle was band-pass filtered (30–500 Hz, 4th order Butterworth) and the response to each stimulus was extracted for analysis using a custom computer program (Matlab, Mathworks). As can be seen in Figure 1, the size of the M, H, and PRM waves dwarfed any background EMG signals and the temporal windows chosen for waveform analysis (gray boxes in the figure) excluded nearly all of the stimulation artifacts. For each H reflex stimulus, if the M-wave elicited during stepping was within ±15% of the standing M-wave at H_max_, then this was considered a match and the corresponding H reflex data was included for further analysis. All PRMRs were included in the analysis. H and PRM reflex amplitudes recorded during stepping were normalized to their corresponding standing reflex amplitude. Reflexes were then averaged within a condition and then across subjects for statistical analysis. We calculated a swing/stance modulation index (MI) for each speed:


(1) $$\begin{array}{l} MI=\left(St-Sw\right)/St \end{array}$$

where MI is the modulation index, St is the stance phase reflex amplitude and Sw is the swing phase reflex amplitude. Therefore, a high or positive modulation index indicates a decrease in swing phase amplitude compared to stance (or more modulation), a MI near zero indicates little reduction in the swing phase amplitude compared to stance and a negative MI indicates the swing phase reflex was larger than the stance phase reflex. Coefficient of variation was also calculated for each subject and condition and then compared across subjects to determine if any significant differences in variability existed between SCI and non-injured groups. For step related EMG patterns, EMG signals were rectified, enveloped (30 Hz. 4th order lowpass Butterworth), extracted from foot contact to the next foot contact per robotic measures (when the position sensors in the robot detect maximal leg orthosis extension AND maximal leg orthosis lowering down to the treadmill), and time normalized using a spline interpolation to 100% of the cycle time.

### Statistical Analysis

Student's *t*-test with unequal variances was used to determine significant difference between stimulation intensities and standing reflex amplitudes between groups. A two-way repeated measure ANOVA (speed, phase) was used to determine statistically significant (α = 0.05) changes in soleus H reflex amplitudes, PRM reflex amplitudes and coefficients of variation. Relationships between variables were assessed using the Pearson correlation coefficient and the Fisher *r*-to-*z* transformation was used to determine statistical differences between correlation coefficients. Reflex modulation indices were compared using an independent samples Student *t*-test with unequal variance if Levene's test of equal variances was significant. Statistical processing was performed using SPSSv21 (IBM).

## Results

### Soleus H Reflex and Lower Limb PRM Reflexes During Standing

The average stimulation intensity needed to elicit the standing soleus H_max_ was greater in the SCI group, ranging from 9.5 to 65 mA than in the NI group with a range of 9–30 mA (*p* < 0.05) (Table 1). Average stimulus intensity needed to elicit H matched soleus PRM reflexes was not different between groups, ranging from 105 to 388 mA for the NI group and 92–360 mA for the SCI group (*p* = 0.22) (Table 1). In 2/10 NI subjects (NI 7 and NI10) and 2/15 SCI subjects (SCI03 and SCI11) the stimulus intensity needed for the H matched PRM reflexes was greater than two times motor threshold (range 2.2–2.5). There was no significant correlation between H and PRM stimulation intensities (NI: *r* = 0.08, *p* = 0.826; SCI: *r* = 0.25, *p* =0.366).

For the soleus H_max_ and the matched soleus PRM reflex, peak-to-peak amplitudes ranged from 0.35 to 2.79 mV for the NI group and 0.13–6.00 mV for the SCI group (Table 2). There was no significant difference between soleus H or PRM reflex amplitudes during standing. The Pearson correlation coefficient between the soleus H and PRM reflex during standing was high for both groups due to matching (Table 3). We were unable to match the soleus PRM reflex to the H reflex in one SCI subject (SCI14: mean H reflex 0.41 mV; PRM reflex 0.13 mV).

**Table 2:** Mean (SD) standing reflex amplitudes.

	**PRM reflex (mV)**					**H reflex (mV)**
**Muscle**	**Quadriceps**	**Adductor**	**Hamstring**	**Tibialis anterior**	**Soleus**	**Soleus**
NI Group	0.41 (0.35)	0.68 (0.36)	1.78 (1.23)	0.30 (0.17)	1.10 (0.78)	1.10 (0.77)
SCI Group	0.67 (0.48)	0.48 (0.34)	1.14 (0.84)	0.43 (0.35)	1.38 (1.24)	1.42 (1.42)
*p*-value	0.141	0.174	0.13	0.27	0.526	0.527

*There were no significant differences between groups*.

**Table 3:** Sol H and PRM reflex correlations for the raw (top) and normalized (bottom) mean.

	**Standing**	**Slow mid St**	**Fast mid St**	**Slow mid Sw**	**Fast mid Sw**
NI	*r* = 0.996	*r* = 0.402	*r* = 0.412	*r* = 0.197	*r* = −0.042
	*p* < 0.01^*^	*p* = 0.249	*p* = 0.237	*p* = 0.586	*p* = 0.908
SCI	*r* = 0.986	*r* = 0.785	*r* = 0.289	*r* = 0.801	*r* = 0.869
	*p* = <0.01^*^	*p* < 0.01^*^	*p* = 0.296	*p* < 0.01^*^	*p* < 0.01
NI		*r* = 0.666	*r* = 0.386	*r* = −0.145	*r* = −0.222
		*p* = 0.036^*^	*p* = 0.270	*p* = 0.689	*p* = 0.538
SCI		*r* = 0.326	*r* = 0.473	*r* = −0.056	*r* = 0.049
		*p* = 0.235	*p* = 0.075	*p* = 0.844	*p* = 0.862

* *Indicates a significant correlation between variables. Also note that some individuals in the SCI group had high raw reflex amplitudes which tend to skew the raw correlations (top)*.

PRM reflexes in other recorded leg muscles, which were elicited simultaneously with the H matched soleus PRM reflex during standing, are shown in Figure 1. During quiet standing, the Ham and Sol muscles produced the largest amplitude PMRs followed by the Add and Quad with the TA having the lowest mean amplitude (Table 2). There was no significant difference between PRM amplitudes across the two groups, NI and SCI, during quiet standing.

### Soleus H and PRM Reflexes During Stepping

**Figure 3:**
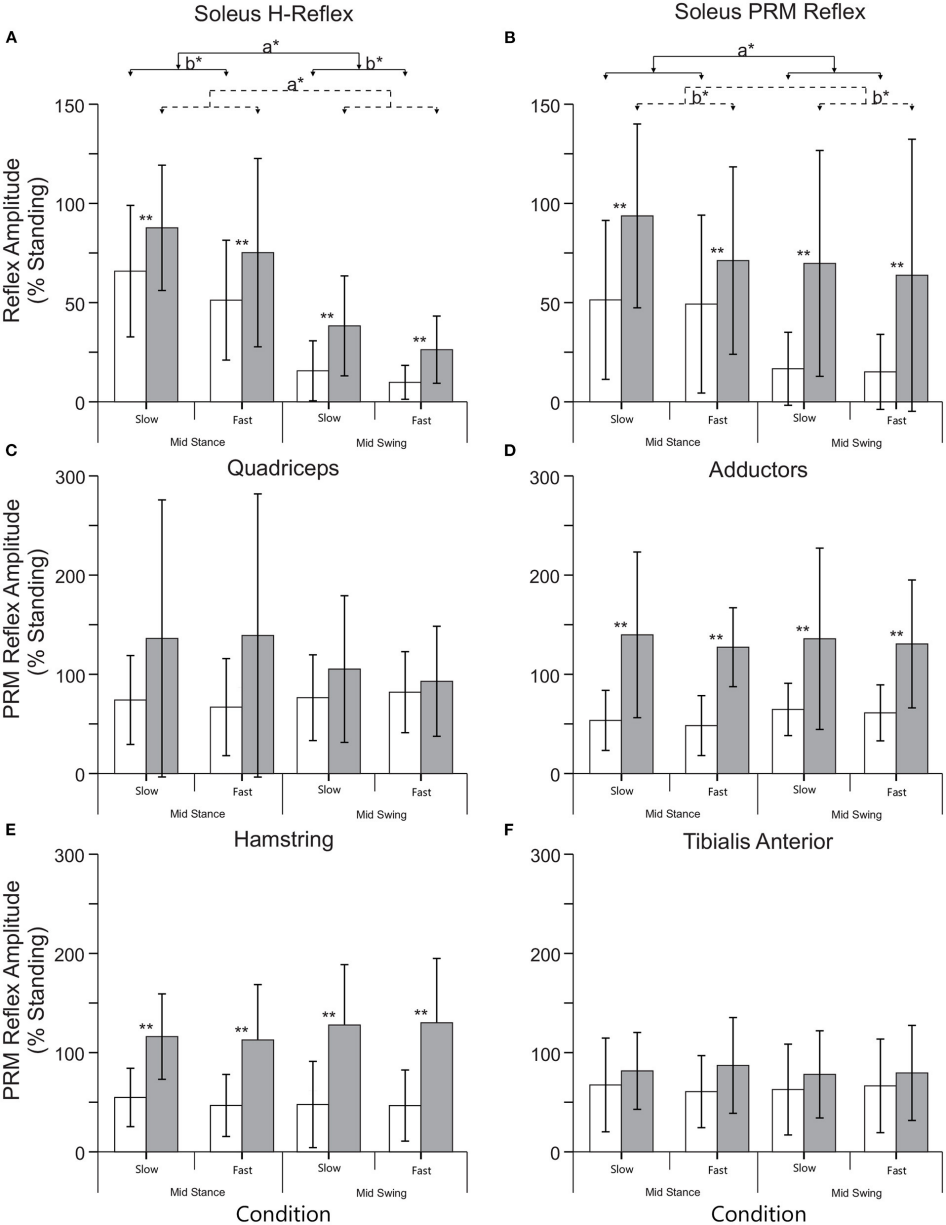
Mean (+SD) reflex amplitudes for each phase (mid stance and swing) and speed (slow: 0.5 m/s and fast: 0.69 m/s) of stepping for the NI (open) and SCI (filled) groups. **(A)** Normalized soleus H reflex amplitude during stepping. **(B)** Normalized soleus PRM reflex amplitude during stepping. **(C–F)** PRM reflex amplitudes from the remaining lower limb muscles during stepping. **Indicates an significant difference between the NI and SCI groups, a* indicates a significant effect of phase, and b* indicates a significant effect of speed. There were no significant differences in speed or phase in muscles other than the soleus.

For both slow (0.5 m/s) and fast (0.69 m/s) stepping speeds, soleus H reflex and PRM reflex amplitudes showed significant step cycle-related modulation for both groups (Figures 3A,B). For the NI soleus H reflexes (Figure 3A), the normalized amplitudes were larger in stance than in swing [F_(1,9)_ = 33.25, *p* < 0.00] and were larger during the slower walking speed than the faster speed [F_(1,9)_ = 5.520, *p* < 0.05] but there was no interaction between speed and phase [F_(1,9)_ = 1.149, *p* = 0.31]. For the NI soleus PRM reflex (Figure 3B), the PRM amplitudes behaved similar to the H reflex, where larger responses were recorded in stance than in swing [F_(1,9)_ = 12.66, *p* < 0.01] without any interaction between speed and phase [F_(1,9)_ = 0.005, *p* = 0.94]. They did not, however, show an effect of speed [F_(1,9)_ = 0.263, *p* = 0.62]. In addition to reflex amplitude changes, the correlation between the NI soleus H and soleus PRM reflex amplitudes during each stepping condition were reduced and significantly weaker during stepping when compared to the correlation during standing (*p* < 0.05 for all conditions, Fisher *r*-to-*z* transformation, Table 3). In total, the percent of the PRM reflex modulation, which was accounted for by modulation of the H reflex ranged from 2 to 44% (*R*^2^) during mid stance and mid swing at both speeds within the NI group.

There was a significant effect of SCI on both soleus H and soleus PRM reflexes during stepping [H reflex: F_(1,23)_ = 6.229, *p* < 0.05; PRM: F_(1,23)_ = 5.728, *p* < 0.05] indicating significantly higher soleus reflex amplitudes during all conditions. There were no interactions between injury and phase and speed [H reflex: F_(1,23)_ = 0.349, *p* = 0.56; PRM reflex F_(1,23)_ = 1.411, *p* = 0.247]. For the SCI group, there was a significant effect of phase on the soleus H reflex [F_(1,14)_ = 31.10, *p* < 0.001], where mid stance reflexes were greater than mid swing, but there was no effect of phase on the soleus PRM reflex [F_(1,14)_ = 2.29, *p* = 0.15]. There was however, a significant effect of speed on soleus PRM reflexes after SCI, where the slow speed had greater reflex amplitudes than the fast speed [F_(1,14)_ = 6.25, *p* < 0.05]. For the SCI group, the correlation between the soleus H and PRM reflex during each stepping condition decreased and were weaker when compared to the relationship during standing (Fisher *r*-to-*z* transformation, Table 3). In total, the percent of the PRM reflex modulation accounted for by modulation of the H reflex ranged from 0.24 to 22% (*r*^2^) during mid stance and mid swing at both speeds with none of the correlations being significant.

### Soleus H and PRM Reflex Modulation Index and Lower Extremity Motor Scores

**Figure 4:**
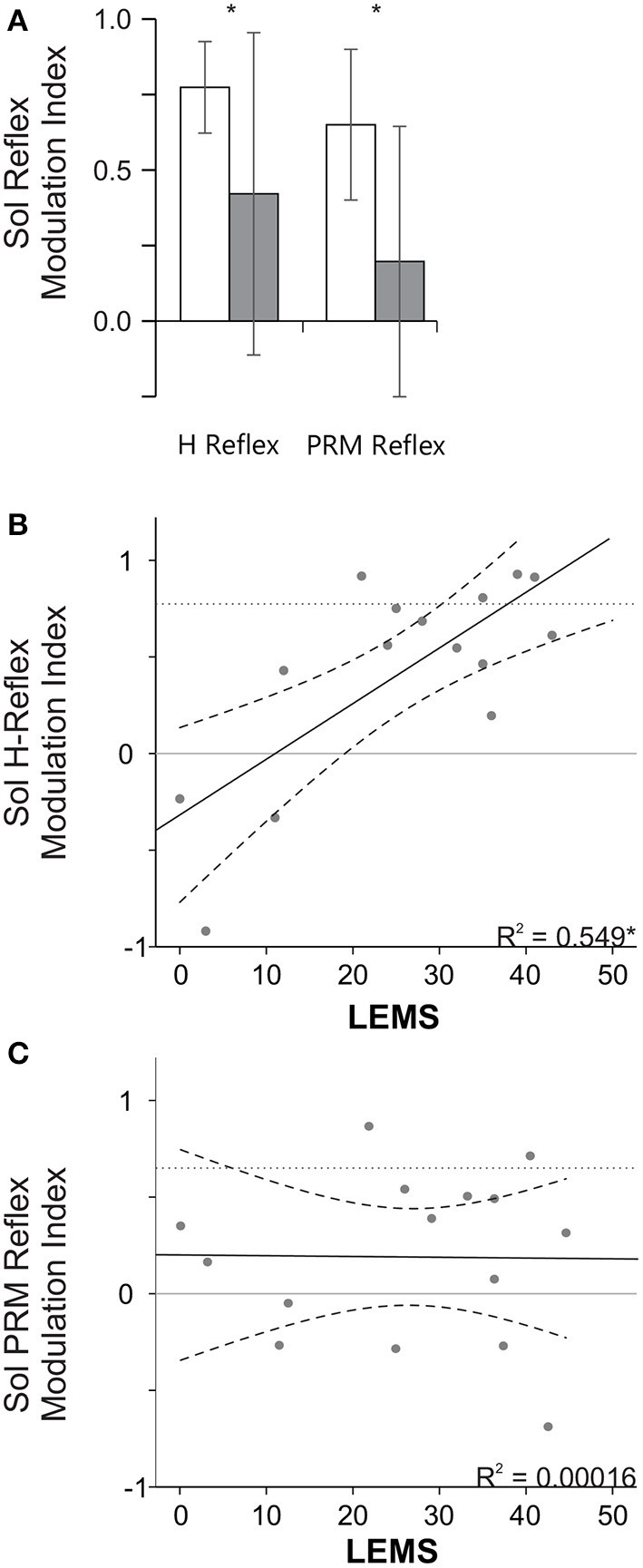
Stance swing modulation index and the relationship to LEMS. **(A)** Mean (+ SD) modulation index for the NI and SCI groups. Note a larger modulation index indicates more step cycle related reflex modulation (i.e., mid stance > mid swing). *Indicates a significant difference between the NI and SCI groups. **(B,C)** Correlation between LEMS and the mean H **(B)** and PRM **(C)** modulation index. *Indicates a significant Pearson correlation (*p* < 0.05). Solid line indicates line of best fit and dashed lines indicate 95% confidence intervals. Dotted horizontal lines represent mean reflex modulation index for the NI group.

To better evaluate modulation between mid-stance and mid-swing, a modulation index (see Equation 1) was calculated for each speed and then averaged across speeds and subjects for each group (Figure 4). The SCI group had a significantly lower modulation index, indicating less step cycle related modulation, compared to the NI group for the soleus PRM reflex (*p* < 0.01) and H reflex (*p* = 0.027, *t*-test). When we examined how the modulation index was related to the lower extremity motor scores (LEMS), we found that there was a significant correlation between the LEMS and the mean soleus H reflex modulation index (*r* = 0.741, *p* < 0.01, Figure 4B) but not for the soleus PRM reflex modulation index (*r* = −0.013, *p* = 0.964, Figure 4C).

### PRM Reflexes From Other Lower Limb Muscles During Stepping

While there was no significant effect of phase or speed on the other PRM reflexes, the SCI group had significantly higher reflex amplitudes throughout stepping in the Add, and Ham muscles but not in the Quad and TA muscles, though generally the means were higher in these conditions as well. Since there was no effect of phase, a modulation index was not calculated for the other lower limb PRM reflex amplitudes, though there were changes in EMG output during stepping as illustrated in Figure 2.

### EMG Prior to Test Stimuli

There was a significant difference in soleus EMG activity prior to test-stimulus delivery during stance and swing phases of gait for both H reflex and PRM reflex testing (Table 4). There was no difference in pre-stimulus EMG during PRM testing in the other muscles.

**Table 4:** Mean (SD) difference between mid stance and mid swing pre-EMG.

	**PRM reflex** **(μV)**					**H** **Reflex** **(μV)**
**Muscle**	**Quad**	**Add**	**Ham**	**TA**	**Sol**	**Sol**
NI mean diff	−5.2 (20.6)	−1.3 (4.3)	1.8 (2.7)	−13.7 (74.7)	15.2 (20.9)^*^	15.5 (21.2)^*^
SCI mean diff	−25.0 (83.2)	−1.0 (2.4)	0.68 (10.0)	−0.86 (17.4)	6.6 (14.9)	4.6 (11.1)

* *Indicates significant a significant difference between mid-stance and mid swing EMG prior (t-test). There was no significant difference between the NI and SCI groups*.

### Variance Across Groups

There was no significant difference in coefficient of variation for the H reflexes and PRM reflexes across conditions (initial standing or stepping), groups (NI or SCI) or reflex types (H or PRM), though data from the stepping conditions tended to have higher variability compared to standing and data from the SCI group tended to have more variability than the NI group.

## Discussion

Our results show that both groups demonstrate step cycle related reflex modulation for the soleus H reflex (Figure 3A) but only the NI group shows step-cycle modulation for the soleus PRM reflex (Figure 3B). For the non-injured subjects, both the soleus H and PRM reflexes were largest during standing, smaller during mid stance but still larger than during mid swing phase during body weight-supported robotic stepping. There was small effect of speed for the NI group where slower speeds generated larger reflexes than the faster speed that is likely due to the timing of stimulus relative to the EMG burst though PRM reflexes did not show this patterning. However, PRM reflex responses from other leg muscles in the NI group did not modulate with stepping phase (mid stance and mid swing) or speed again likely due to timing of stimulus relative to EMG activity (Figure 2) and no difference in mean EMG activity prior to stimulus (Table 4). Subjects with SCI produced soleus H and PRM reflex responses that were larger than the NI group but only the H reflex demonstrated significant step cycle related modulation. In addition, subjects with SCI had less step cycle related reflex modulation (lower modulation index) compared to the NI group (Figure 4A) but when we examined the relationship between reflex modulation and lower extremity motor scores, those subjects with higher motor scores had better H reflex modulation (Figure 4B). The same was not true for the PRM reflex (Figure 4C). Overall, there was little correlation between the H and PRM reflexes during each stepping condition for either group (Table 3).

### Soleus Reflex Modulation

Similar to previously published data, step cycle related modulation of the H (6–8) and PRM reflexes (9, 12, 15) was observed (Figures 3, 4). However, while both reflexes exhibit step cycle related modulation, our results suggest that PRM reflexes modulate differently than H reflex modulation resulting in the poor correlation between H and PRM reflexes (Table 3). Thus, the average H reflex amplitude for a given individual and condition did not accurately predict the corresponding value of the PRM reflex amplitude (Table 3), suggesting the PRM reflexes may be more complex than the traditional H reflex.

There are several possible reasons soleus PRM reflex modulation may be different than the soleus H reflex. First, for the PRM reflex, it is possible, although unlikely, that there is an M-wave or direct efferent activation component included in the PRM reflex waveform. For example, Knikou found that PRM reflex responses do not undergo homosynaptic depression (14) suggesting that perhaps there was an efferent component to the PRM reflex behavior in that study. Though their stimulation setup was different than outs om that they positioned large electrodes over the iliac crests potentially generating anodal stimulation of the femoral nerve, direct efferent activation is still a potential confounding factor. Efferent stimulation (M waves) however seems unlikely for several reasons. First, PRM reflexes do demonstrate modulation from standing at mid stance and mid swing. Therefore, during standing and mid stance any M-wave component is not the largest component of the signal (since swing phase responses were generally the smallest if the afferent input is completely inhibited this could potentially be a residual efferent component). Second, work from epidural cauda equina stimulation (4) and modeling results (18) suggest that higher stimulation intensities, ~2–2.5× threshold, were necessary to begin to activate efferent ventral roots. Based on our results only 2/10 NI and 2/15 SCI subjects reached this level, yet, each of these subjects demonstrated at least a 50% reduction in reflex amplitude during swing phase of stepping compared to standing, suggesting that if an M-component was present it was not a major contributor to response amplitude.

Thus, a more plausible explanation for the different modulation of PRM reflex is that it is derived from more heteronymous afferent input with less temporal dispersion than is brought by tibial nerve stimulation. Given, that stimulation of the PRM reflex is thought to activate primarily dorsal roots before entering the dorsal horn of the spinal cord, then this heteronymous input from multiple dorsal roots would project simultaneously across multiple segmental levels and complex interneuronal circuitry. For instance, while tibial nerve stimulation depolarizes principally homonymous large 1A afferents compared to the mixed population of afferents (Ia, IIb, A-beta) possibly activated in the PRM reflex. Therefore, different reflex pathways (e.g., H reflex and crossed-extension-reflex) may be activated to similar degrees with this multi-segmental input and the interactions of these inputs are unknown at this time. In addition, afferent axons stimulated to elicit a soleus H reflex have different conduction times creating some temporal dispersion when traveling from the popliteal fossa to the first synapse in the spinal cord. This could potentially allow activation of some inhibitory or excitatory interneurons before summation of inputs onto the motoneuron altering its decision to fire. However, afferent input eliciting a PRM reflex would be much more synchronous given its close proximity to the cord, suggesting that some interactions (e.g., activation of a reciprocal inhibitory interneuron by a faster conducting fiber) may not have time to occur and may leave the motoneuron in a slightly different state of excitability than is expected in the H reflex.

### Impact of Injury on Reflex Modulation

In terms of SCI, we found that individuals with a spinal cord injury have significantly higher reflex amplitudes, as a percentage of their standing reflex, during stepping when compared to the non-injured subjects and that there is less step cycle related reflex modulation (Figure 4A). These larger reflexes are consistent with the hyperreflexia aspects of spasticity an upper motoneuron syndrome (19, 20) where the segmental spinal circuitry is in a higher state of excitability. The PRM reflexes may be further complicated by the various states of excitability for heteronymous input. Similar to previous results (21, 22), we found that after incomplete SCI the H reflex was reduced during mid swing compared to mid stance phase of stepping (Figures 3, 4) but we did not see the same modulation in the PRM reflexes. In general the results were similar to those seen by Dy et al. (12). Generally, the reflex amplitude patterned the EMG activity during the step, though in Dy et al. subjects had more complete injuries and less supraspinal excitation, suggesting that if the muscle activation was inappropriate for the phase of stepping then reflex amplitude or modulation would also be inappropriate.

SCI complicates interpretation of the PRM reflexes. Based on our data, it appears that the ability to appropriately modulate H reflexes but not PRM reflexes is related to the LEMS (Figure 4B), which could be an indicator of the degree of volitional control and supraspinal regulation of excitability after SCI. These results suggest that in humans H reflex modulation, as well as voluntary muscle activity, has a large supra-spinal component (5). However, the fact that PRM reflexes show such little correlation to LEMS may be due to the broad spectrum of afferent input types and the large number of neurons activated from multiple segments arriving at an injured spinal cord at varying levels of excitability. These results seem to agree somewhat with the larger idea of spasticity as an upper motoneuron syndrome with not only muscle stretch inputs In an altered state of excitability but other afferent inputs are dysregulated after SCI as well (19).

### Clinical Implications

In the context of locomotor training, reflex modulation becomes an important variable. For instance, Thompson and Wolpaw found that the ability to down regulate H reflexes lead to improved walking after SCI in spastic patients (although up regulation of H reflexes in hypo-reflexic patients did not improve stepping) (23, 24). Therefore, with locomotor training, it may be that individuals who have more appropriate step cycle related modulation have a better chance of improving walking function with training. Given that we generally know that persons with a less severe injury (high LEMS) tend to do better in locomotor training (25) this may reinforce the idea that the ability to coordinate muscle activity throughout the step cycle is a prerequisite for demonstrating improved stepping and that better methods are needed to quantify post-SCI motor control patterns. These methods could include both the H and PRM reflexes as biomarkers for therapeutic effects of interventions in SCI where normalization of the H and PRM reflex modulation would represent recovery of more normal spinal circuit function or better supraspinal regulation of that circuitry.

## Conclusions

We investigated whether H and PRM reflexes were modulated similarly during stepping in individuals with and without SCI. We found that H and PRM reflexes did modulate with the step cycle in the NI group but that only the soleus H reflex modulated with the step cycle in the SCI group. However, after SCI, H reflex modulation was not as robust. We suspect that the inability to provide step cycle related modulation is due to altered supraspinal regulation of segmental excitability as the ability to modulate the H reflex appears to be related to the LEMS an indicator of volitional control. In this regard, PRM reflexes clearly modulated differently than H reflexes. Thus, in future locomotor studies, it could be beneficial to assess an individual's ability to modulate reflexes during stepping and see how this modulation changes with training.

## Data Availability Statement

The raw data supporting the conclusions of this article will be made available by the authors, without undue reservation.

## Ethics Statement

The studies involving human participants were reviewed and approved by Shepherd Center IRB. The patients/participants provided their written informed consent to participate in this study.

## Author Contributions

BF participated in experimental design, data collection, data analysis, and article preparation. JB participated in subject recruitment, data collection, and article preparation. WM participated in data collection, data analysis, and article presentation. KT participated in winning funding for the project, experimental design, data collection, data analysis, and article preparation. All authors contributed to the article and approved the submitted version.

## Funding

This work was supported by VA RR&D 5I01RX000417.

## Conflict of Interest

The authors declare that the research was conducted in the absence of any commercial or financial relationships that could be construed as a potential conflict of interest.

## Publisher's Note

All claims expressed in this article are solely those of the authors and do not necessarily represent those of their affiliated organizations, or those of the publisher, the editors and the reviewers. Any product that may be evaluated in this article, or claim that may be made by its manufacturer, is not guaranteed or endorsed by the publisher.
