# Supplementary material for: Soleus H and Lower Limb Posterior Root Muscle Reflexes During Stepping After Incomplete SCI
Source: Front Rehabil Sci. 2022 May 13;3:789333. doi: 10.3389/fresc.2022.789333 (PMC9397667; doi:10.3389/fresc.2022.789333)
Supplement: Supplementary file 1 [file Data_Sheet_1.PDF]

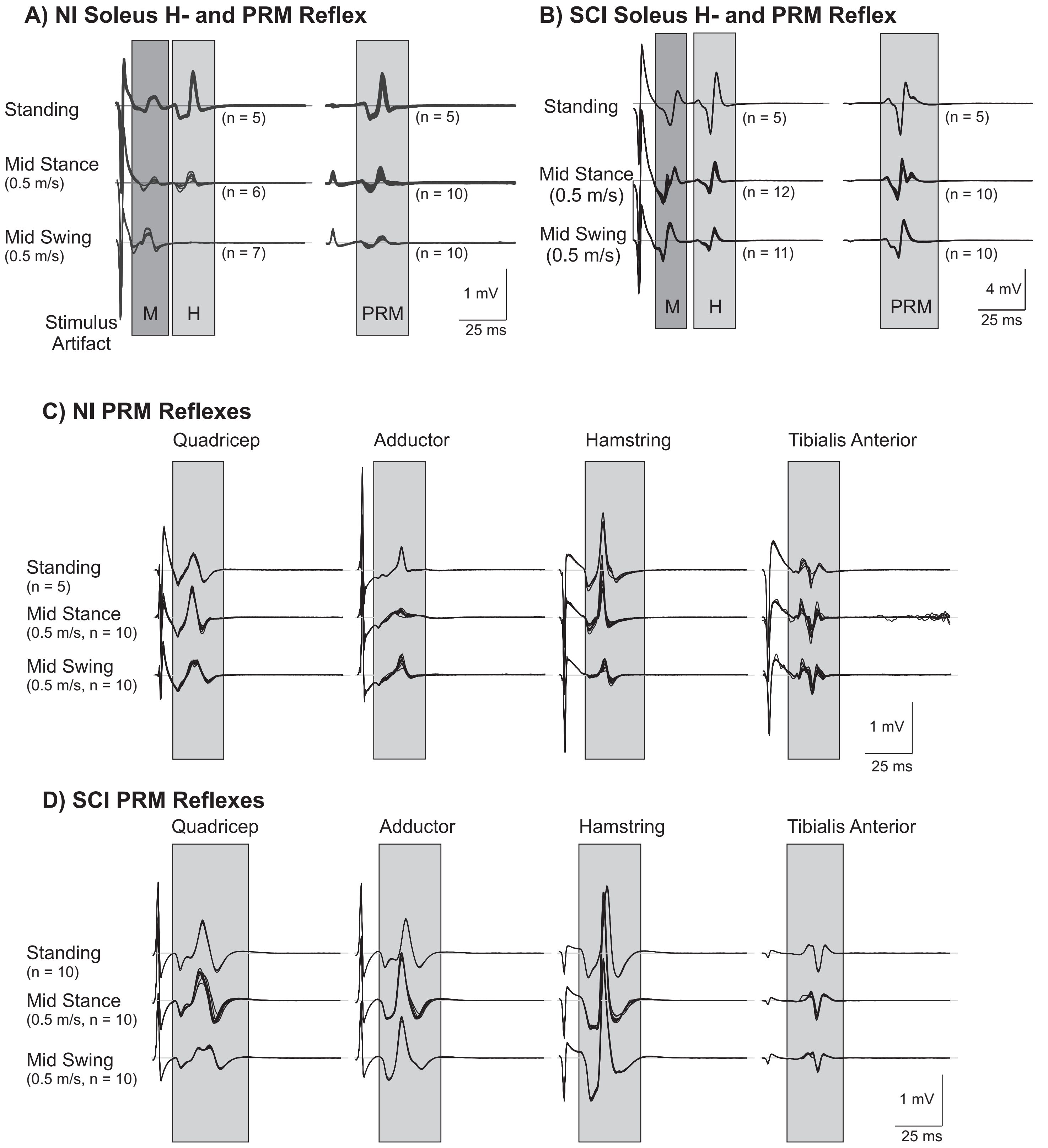

Figure 1: Representative soleus H- and lower limb posterior root muscle (PRM) Reflexes. A,B) Soleus H- and PRM reflexes from a subject without injury (NI#, A) and a subject with a spinal cord injury (SCI#, B). Note the mid-swing reduction of H and PRM in the soleus is absent in the SCI subject. C,D) PRM reflexes from other lower limb muscles: hip adductor (Adductor), rectus femoris (Quadriceps), biceps femoris anterior (Hamstring) and tibialis anterior from the same subject without injury (C) and the same subject with SCI (D). Gray areas highlight the approximate time frame of the H- and PRM reflexes.

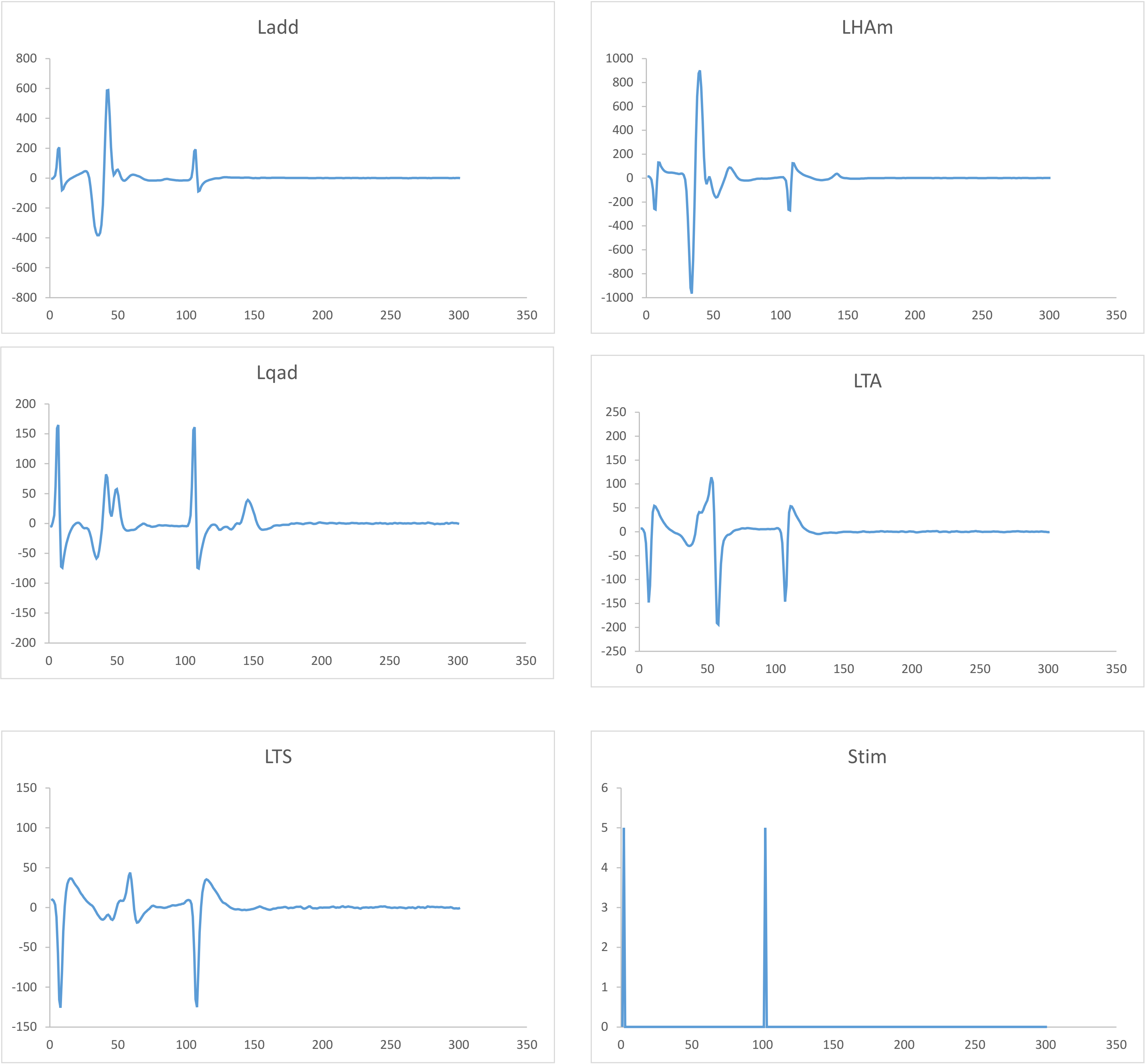

Paired pulse graphs at common motor threshold with 50 ms interpulse interval (time above is in samples, rate is 2KHz). **We did not do paired pulses at PRMR = Max H.**

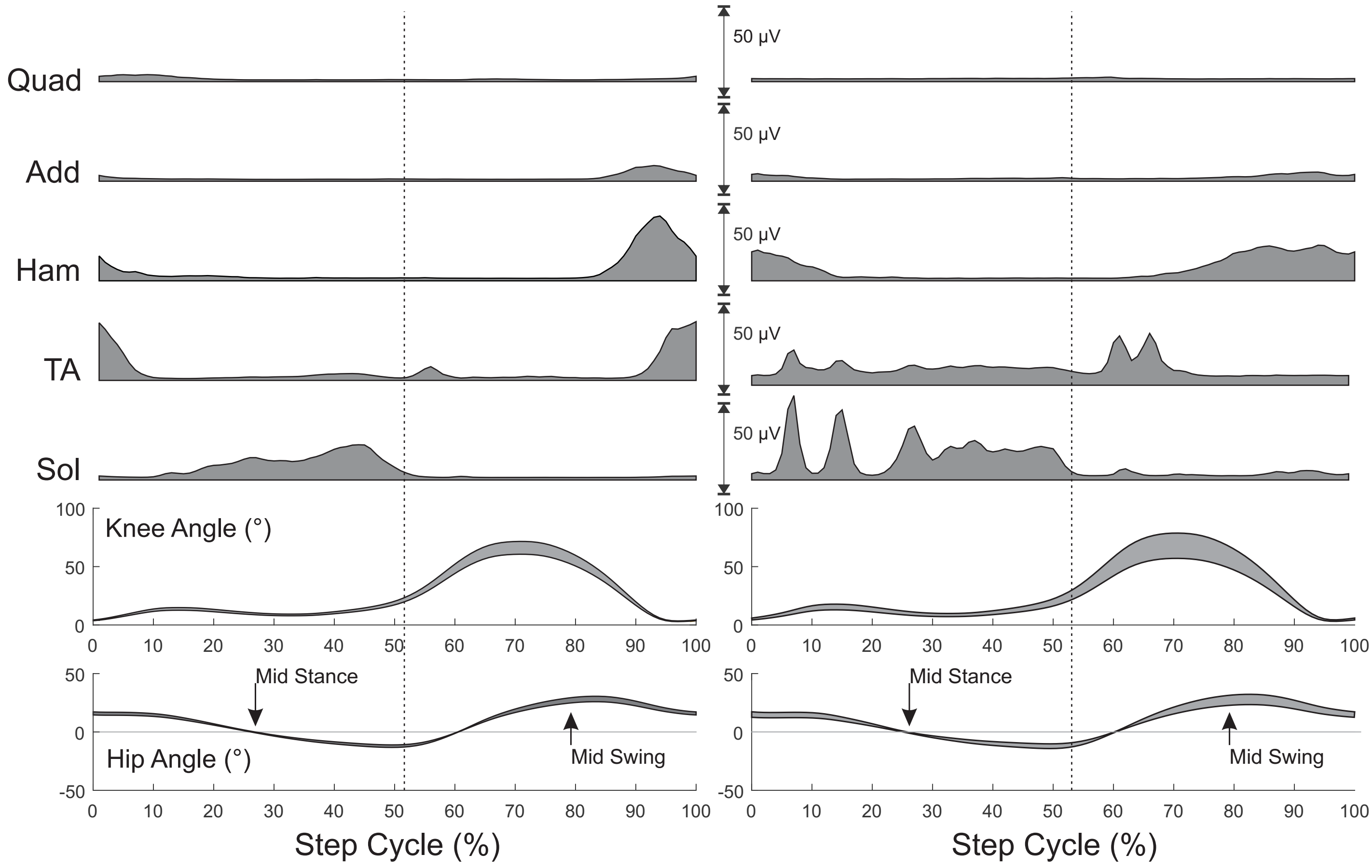

Figure 2: Muscle activity and joint angles during stepping for a subject without injury (Left) and a subject with SCI (Right). All values are time normalized to 100% of the step cycle. Muscle activity is the mean activity at each 1% of the step cycle over 25 steps prior to eliciting reflexes. Joint angles represent mean (± SD) during the same steps. Arrows indicate mid stance and mid swing phase of stepping when reflexes would be triggered. Mid stance and mid swing hip joint angles were established during this initial stepping period. Vertical dashed lines represent the transition from stance to swing.

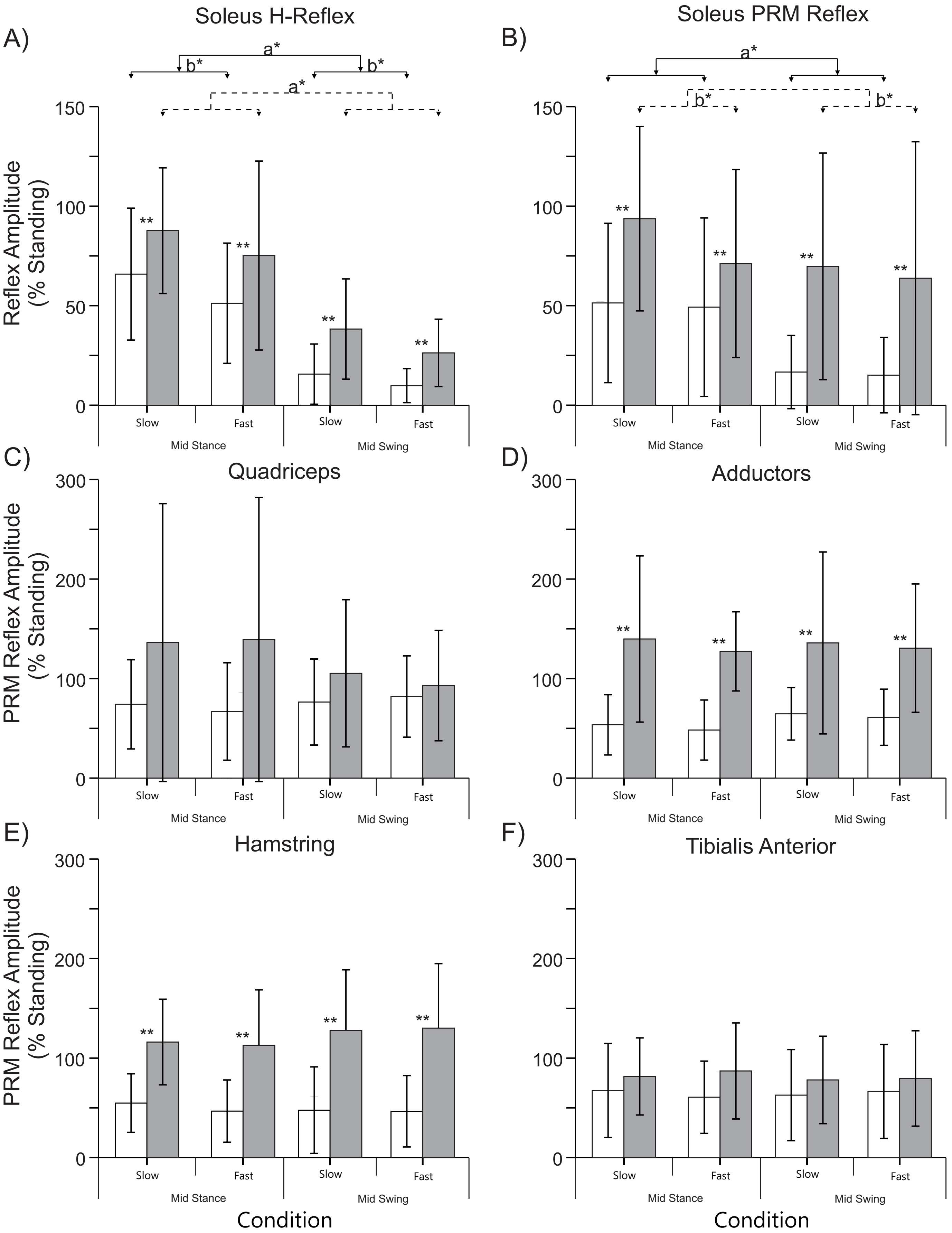

Figure 3: Mean (± SD) reflex amplitudes for each phase (mid stance and swing) and speed (slow: 0.5 m/s and fast: 0.69 m/s) of stepping for the NI (open) and SCI (filled) groups. A) Normalized soleus H-reflex amplitude during stepping. B) Normalized soleus PRM reflex amplitude during stepping. C-F) PRM reflex amplitudes from the remaining lower limb muscles during stepping. Note ** indicates an significant difference between the NI and SCI groups, a* indicates a significant effect of phase, and b* indicates a significant effect of speed. There were no significant differences in speed or phase in muscles other than the soleus.

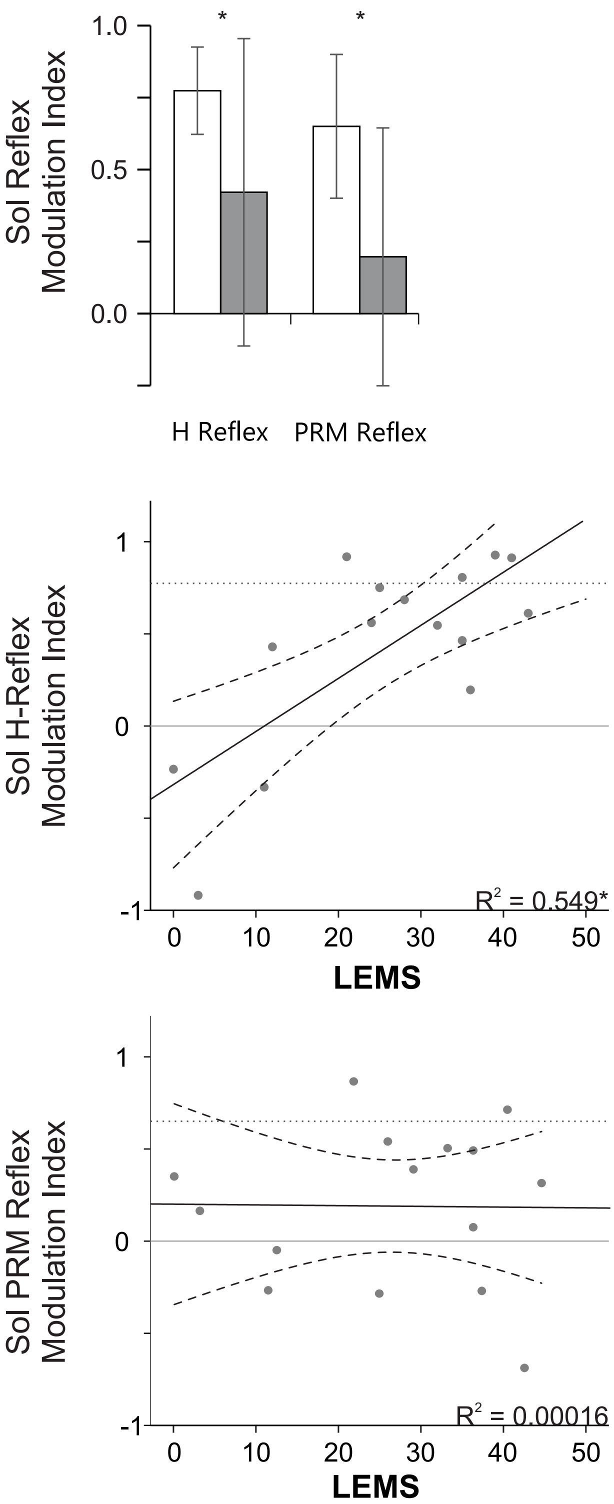

Figure 4: Stance swing modulation index and the relationship to LEMS. A) Mean (± SD) modulation index for the NI and SCI groups. Note a larger modulation index indicates more step cycle related reflex modulation (i.e. mid stance > mid swing). * indicates a significant difference between the NI and SCI groups. B,C) Correlation between LEMS and the mean H- (B) and PRM (C) modulation index. * indicates a significant Pearson correlation ($p < 0.05$). Solid line indicates line of best fit and dashed lines indicate 95% confidence intervals. Dotted horizontal lines represent mean reflex modulation index for the NI group.
